# How Observed Personality Traits in (Mildly) Depressed Adolescents Relate to Nonverbal Responses of Peers

**DOI:** 10.1007/s10578-024-01669-3

**Published:** 2024-02-12

**Authors:** Marry Schreur, Yolanda van Beek, Roos Hutteman

**Affiliations:** Department of Developmental Psychology, Faculty of Social and Behavioral Sciences, Utrecht University, Utrecht, Netherlands

**Keywords:** Observed personality traits, (mild) Depression, Adolescence, Nonverbal behaviors, Gender differences

## Abstract

Depression in adolescence is related to negative social responses. Previous studies indicate that negative responses precede, co-occur and follow depressive episodes, indicating that more stable characteristics of depressed(to-be) adolescents may trigger such responses. This study examines whether personality traits, as observed in behavior, mediate or moderate responses of peers towards (mildly) depressed adolescents. Nonverbal responses of peers were observed during two short semi-structured interactions, one with a (mildly) depressed partner and one with a nondepressed partner, matched for age and gender. Personality traits of partners were observed. Results show that peers responded more negatively towards (mildly) depressed partners. Personality traits *moderated* the link between depression and peer responses. In general, but particularly for (mildly) depressed girls, neuroticism strengthened the link between depression and negative peer responses, while expressivity diminished the link between depression and negative peer responses. For boys, small and counterintuitive moderation effects were found which merit further research.

**Supplementary Information:**

The online version contains supplementary material available at 10.1007/s10578-024-01669-3.

## Introduction

According to the World Health Organization (WHO), depression affects 280 million people worldwide and suicide is the fourth leading cause of death in adolescents and young adults [1]. However, as common as depression has become, there is also the conception that people with depressive characteristics are perceived in a negative way. There is a tendency to avoid, ignore, or treat depressed persons less friendly than others [2]. These negative reactions are especially significant during adolescence, when friendships and relationships are known to be important for wellbeing and the development of identity [2]. Depression has been shown to increase between the ages of 13 and 16 years, most notably for girls, and the social problems and mood disturbances that adolescents experience may increase their vulnerability for a recurring depression in adulthood [3]. It is not yet clear, however, what characteristics of (mildly) depressed adolescents might trigger these negative reactions. Do peers negatively respond to behaviors that result from a depressive mood, or are they responding to less popular behavioral characteristics that the depressed adolescent displays more generally, independent of the depressive episode?

Examining the relations between depression and social problems during adolescence reveals a well-established link. The quality of communication is rated lower with depressed adolescents and they are perceived as less socially capable and are less likely to be rated as popular [2, 4–6]. Furthermore, not only major depression, but also mild or initial depressive symptoms were shown to erode social support in adolescence [7]. A study focusing on short interactions revealed that adolescents show less positive and more negative nonverbal behaviors towards (mildly) depressed peers [8, 9].

In addition to the concurrent link between depression and social problems, literature has shown that social problems, such as relational victimization and negative experiences in close relationships, are also predictive of depression, especially for girls [2, 10–13]. Furthermore, lack of peer support and social adjustment were shown to be chronic risk factors for depression, in that depressed adolescents rated them lower before, during and after a depressive episode than non-depressed adolescents [14]. It was found that for girls, the differences in nonverbal responses towards depressed adolescents were already present before adolescents became (mildly) depressed [9]. It does not suffice to say then, that adolescents are simply reacting towards ‘depressed’ behaviors, as social problems precede and are predictive of depressive characteristics, as well as remain after. This suggests that there are more stable characteristics present within the individual that influence their social behaviors and trigger the negative peer responses prior to, during and after a depressive episode.

Personality is a plausible candidate for stable characteristics that are present over time and that are important in social relationships. Literature shows that there are clear links between personality [15] and depression. Both in clinical and general populations high levels of negative affect and low levels of positive affect have been found to characterize depression [16] and negative affect and positive affect have in turn been strongly linked to neuroticism and extraversion respectively [17]. High levels of neuroticism as well as low levels of extraversion have consistently shown to be associated with depressive symptoms in studies comparing clinical to general population adults [18]. Extraversion has been shown to be a protective factor for depression [19], while low extraversion is a risk factor for first onset and recurrent depression for adolescent girls [20]. Neuroticism is associated with depression for early adolescents [21].

When considering personality traits as the stable characteristics that might underlie or influence the negative responses depressed adolescents experience, it is necessary to understand how personality traits can be perceived. Though it is well known that there are strong cognitive aspects to personality, such as cognitive strategies or interpretation of loci of control, personality also contains a strong behavioral aspect, in that people evoke emotions or actions from other people by what they do. But how do we then perceive personality through behaviors? According to the lens model, people use a ‘lens’ for indirect perception and judgment [22]. With just a limited set of cues, perceivable attributes, and nonverbal behaviors, people will make judgments about the personality traits of their interaction partner [23, 24]. Even for short interactions, the perceptions or first impressions of others are lasting and determining for successful social interactions [25]. Extraversion and low neuroticism have been shown to be associated with more likeability and popularity [26]. Some personality traits are also more easily observable than others [27] and may play a greater role in determining a first impression. For example, extraversion can be expressed through observed expressivity and dominance, and when these are reflected during interaction, a partner will consider the interaction as a positive experience [24]. People with high levels of agreeableness display more positive other-oriented behaviors, leading to their interaction partners perceiving the interaction as being of higher quality [28, 29]. Alternatively, neuroticism corresponds with communication apprehension and shyness and predicts nervousness in interaction [30, 31].

The present study will assess personality using observations and investigate whether the negative peer responses that depressed adolescents receive can be explained or influenced by their personality traits, as displayed in their behavior during interaction. Do adolescent peers react more negatively towards a depressed versus a nondepressed partner because depressed adolescents show more negative and less positive personality traits during interaction (*mediation model*)? Or is there no difference in observed personality traits between depressed and nondepressed adolescents, but are the negative responses of peers strengthened or weakened by the observed personality traits of the depressed peers (*moderation model*)?

It is expected that peers show more negative responses towards (mildly) depressed than nondepressed partners. In the mediation model it is expected that the more negative response of peers towards depressed adolescents is (partly) explained by more negative observed personality traits in depressed partners. It is expected that higher observed levels of neuroticism as well as lower levels of agreeableness and extraversion will be observed in depressed partners, which will (partially) account for the difference in peer responses towards depressed versus nondepressed partners.

In the moderation model, it is expected that higher perceived neuroticism may exacerbate or strengthen the negative responses of peers towards (mildly) depressed partners, while higher observed agreeableness and higher extraversion may protect from the negative responses, thereby decreasing negative peer responses.

When examining the relations between depression, personality and nonverbal peer responses in adolescence, the role that gender may play cannot be ignored. Girls have been found to behave in a more other-oriented and friendlier manner [8, 9], score higher in agreeableness and neuroticism [32, 33] and also show more depressive characteristics in adolescence [10]. Studies have shown that girls are expected to show more communal traits, those that focus on relationships between people (e.g., agreeableness), while boys are expected to show more agentic traits, which are more focused on achieving goals (e.g., dominance) [34]. The present study will examine gender differences in whether personality traits explain *(mediation model)* or influence *(moderation model)* the negative responses towards depressed adolescents. It is expected that there will be differences in the role specific personality traits play for girls and boys in accounting for the relationship between depression and negative responses. For instance, girls generally display higher levels of agreeableness, in line with gender-specific display rules [35]. When this does not occur, their peers may perceive them to be less agreeable and respond more negatively, as compared to boys. For boys, it may be more problematic or less accepted to display higher levels of neuroticism, leading to more negative responses from their peers, while for girls these consequences might not be as problematic. And in line with social expectations regarding agentic traits, lack of dominance for boys may lead to more negative responses compared to girls.

To conclude, the present study examines two research questions: (1) Are negative nonverbal responses of peers towards depressed (as compared to non-depressed adolescents) mediated by differences in the observed personality traits? Are these mediating effects different in boys and girls? And/or (2) are the negative nonverbal responses of peers towards depressed (as compared to non-depressed adolescents) moderated by differences in observed personality traits? Are these moderating effects different in boys and girls?

## Method

### Participants

Participants were selected from a larger longitudinal study in the Netherlands (see [36]) that included 606 children from 2 large schools with locations in several villages and cities in the central part of the Netherlands. Participants in the present study were in years 1 through 4 and taken from all 3 levels of secondary school in the Netherlands (lower/intermediate vocational education (VMBO), intermediate/higher vocational education (HAVO), pre-scientific (college preparatory) education (VWO/gymnasium). This study was approved by the medical ethical committee of Utrecht University, in the Netherlands, and confidentiality of the collected data was ensured. In the present study, a total of 195 adolescents, including 102 girls (52%) and 93 boys (48%), who interacted in 166 conversations. Adolescents were in grades 1 to 4 of secondary school, which corresponds to 12 to 18 years of age (*M* = 14.76, *SD* = 1.20).

### Design

All adolescents from the total sample (see [36]) who were above the (sub)clinical cut-off score on the CDI were selected to participate in the present study and were matched for gender, age and school-level with 2 non-depressed controls. Adolescents first completed the Children’s Depression Inventory (CDI, [37]), following which they were classified as non-depressed or (mildly) depressed. Adolescents with CDI scores below the cut-off of the 50th percentile (i.e. ≤8) were classified as control/non-depressed adolescents (*M* = 2.66, *SD* = 1.81), and adolescents with CDI scores above the cut-off of the 75th percentile (i.e. ≥11) were classified as (mildly) depressed (*M* = 16.69, *SD* = 4.85). The 75th percentile was used as indication of (mild) depression in order to increase sample size, as less adolescent boys fell above the (sub)clinical cut-off CDI score. The below 50th percentile for the control group was chosen to create a clear distinction between the (mildly) depressed and non-depressed adolescents.

A non-depressed adolescent peer conducted two 5-minute video-taped conversations, one with a fellow non-depressed adolescent and one with a (mildly) depressed adolescent, who were matched for age, sex, school, and education level. The conversations were semi-structured; the adolescents were given the task to discuss a relevant social dilemma (e.g., “*what would you do if you saw your close friend steal something from a store?*”) until they agreed upon the best solution. If adolescents completed this within five minutes, they were additionally asked to compile and agree upon a top-five list (e.g., favorite movies, teachers, courses, etc.). Adolescents were partnered with non-classmates and not friends, so that their responses would be based on what they observed in that interaction and not on previous relational history. Before the interaction, it was checked whether the adolescents knew each other and if this was the case, then new pairings were made. Participants were unaware of the specific aims of the study during recordings (they were informed afterwards). Considering that adolescents were only selected to participate in the video-taped conversations based on complete information regarding demographics and depression scores and all video-taped conversations were subsequently coded for nonverbal behaviors and personality, there was no missing data.

As not all personality traits are readily observed in short interactions, the present study considers neuroticism, extraversion (subdivided in expressivity and dominance) and agreeableness (see Appendix for how these personality traits were defined). Personality traits of the mildly depressed and the nondepressed control partners and nonverbal responses of a (nondepressed) peer towards both partners, were objectively observed and coded by different coders who did not know which adolescents were (mildly) depressed. Depressive symptoms were measured by means of self-reports, thereby avoiding shared method variance.

### Measures

#### Depressive Symptoms

Depressive symptoms were measured using a Dutch version [38] of the CDI [37]. Reliability of this scale was good (Cronbach’s α = 0.81).

#### Behavioral Observations of Peer Responses

Participants were aware that the conversations were videotaped, however they were unaware that their nonverbal behavior was being observed. Nonverbal behaviors were chosen as they occur more naturally and spontaneously during interaction and are less regulated, thereby presenting a more honest and natural response to partners during interaction [23]. Inter-rater reliability between trained coders was calculated using Cohen’s kappa (range 0.61-0.92, mean 0.74). Coders were unaware of the depression scores of the participants that they coded.

##### Other-Oriented Behaviors

Based on [8]; gazing, talking, backchanneling and smiling were coded using Observer XT (version 11); see Appendix A for further details. All behaviors were scored without sound, except for talking. The interaction was coded multiple times, for each behavior separately.

- *Gazing*: directing the eyes towards the face of the conversation partner. Gazing as percentage of time talking (*gazing while talking*) and gazing as percentage of time listening to partner talk (*gazing while listening*) were calculated and included.
- *Back-channeling*: nodding or vocal utterances, such as ‘yes’ or ‘hmm-hmm’, that indicate that the participant shows interest or confirmation of the partner without interrupting the partner. This was included as the frequency of back-channeling while listening to partner talking.
- *Smiling*: smiling or laughing while listening, included as frequency per minute.

##### Negative Hehaviors

The negative behaviors were measured by coding behaviors and postures that have been mentioned in literature regarding behavioral observations in (mildly) depressed adolescents [9], depressed adults [39–42] and general literature regarding negative behaviors in children and adults [43, 44]; see Appendix A for a more detailed descriptions of behaviors and rating scales.

- *Negative facial expressions*: measured by coding facial expressions that indicated negativity, rejection, or contempt (e.g., frowning, blank expression, yawning). These were rated on a 5-point Likert scale ranging from not/barely occurring to occurring relatively often.
- *Signs of disinterest*: measured by coding the following behaviors and postures: cues of boredom/disinterest in movements (e.g., explicitly chewing gum, shrugging shoulders), cues of boredom/disinterest in posture (e.g., slouching, slightly turned away position), and lack of intonation (e.g., monotonous speech). These behaviors and postures were rated on 5-point Likert scales (ranging from not/barely occurring to occurring relatively often) and taken together, as each only rarely occurred and behaviors had similar meaning. The mean score of these sub-behaviors/postures was used in analyses.
- *Signs of discomfort*: measured by coding the following behaviors: facial restlessness (e.g., rubbing lips, biting cheeks), tensed body position (e.g., stiff posture, chin on chest), and nervous body touching (e.g., scratching, fidgeting with hair or clothes). These behaviors and postures were rated on 5-point Likert scales (ranging from not/barely occurring to occurring relatively often) and taken together, as each only rarely occurred and behaviors had similar meaning. The mean score of these sub-behaviors/postures was used in analyses.

#### Personality Observations of the Partner Adolescents

Personality traits of both the depressed and non-depressed partners with whom the same peer interacted were observed during conversation, specifically the personality traits: agreeableness, neuroticism and extraversion. As extraversion can be observed as two constructs, expressivity and dominance [24], the present study included expressivity and dominance separately in the analyses, as being indicative for extraversion. Trained coders, different than those that coded nonverbal behaviors, were given a short definition of the personality traits examined within the present study (e.g., “*agreeableness is the measure of good-naturedness, kindness, and warmth a person displays…*”) and were asked to rate participants on a 5-point Likert scale (e.g., 1 = *not agreeable/disagreeable*, 5 = *very agreeable*). See Appendix B for a full description of the definitions and rating scales used [15, 45]. Inter-rater reliability was substantial to good, indicated by Cohen’s kappa ranging between 0.65 and 0.84.

### Data Analytic Strategy

All analyses were examined using multilevel models in Mplus 7.2 [46] to account for the multilevel structure in the data, as the same adolescent had interactions with both a non-depressed and a depressed peer.

To investigate the mediating effects of personality traits in the relationship between nonverbal behaviors in interactions with depressed or non-depressed adolescents, multilevel mediation analyses were conducted, using analysis Type = complex and the Maximum Likelihood estimation with Robust standard errors (MLR estimation), which is robust to the non-normality and non-independence of observations. Models were specified per personality trait examining both the direct and indirect effects on the 7 (clusters of) nonverbal behaviors. The direct effects of interacting with a depressed peer (coded 0/1) on the personality trait rating and the nonverbal behaviors were specified, as well as the direct effects of the observed personality trait on the nonverbal behaviors. Indirect effects were modeled using the MODEL INDIRECT command, specifying that the personality trait of the depressed partner mediated the relationship between interacting with a depressed partner and the nonverbal peer responses. Subsequently models were specified separately for girls and boys, to examine whether there were differences in the direct and indirect effects based on gender. Sample sizes were not large enough to test the gender differences in one model due to complexity.

To investigate the moderating effects of personality traits on the relationship between nonverbal responses to depressed or non-depressed adolescents, multilevel moderation analyses were conducted, using the analysis Type = complex and the MLR estimation. Separate models were specified per personality trait, modeling the direct effects of interacting with a depressed peer (coded 0/1) and the personality trait on the nonverbal responses, as well as modeling the effect of the interaction term on the nonverbal responses. Interaction effects were demonstrated using the MODEL CONSTRAINT command. Models were subsequently specified for girls and boys separately, to examine the gender differences in both the direct and interaction effects.

## Results

### Preliminary Analyses

To examine validity of the chosen nonverbal behaviors, 171 participants (114 dyads) indicated how satisfied they were with the conversation directly after the recording, where both participants in the dyad answered four questions on a 10-point scale (‘*How satisfied are you with the result of the discussion?*’, ‘*How much did you like the discussion with this person*?’, ‘*How much do you think the other liked the discussion with you?*’, and ‘*How well did you manage to come to an agreement?*’). All other-oriented behaviors, except backchanneling, correlated significantly positive with the sum of own satisfaction scores and/or the sum of partner satisfaction scores with correlations ranging from 0.16 to 0.23, *p* < .05, for partner satisfaction scores, and from 0.27 to 0.32, *p* < .05, for own satisfaction scores, irrespective of scores on the CDI. Negative behaviors correlated significantly negatively with own and/or partner satisfaction, with correlations ranging from − 0.13 to − 0.24, *p* < .05, with partner satisfaction, and − 0.27 (only negative facial expressions), *p* < .05, with own satisfaction. Other-oriented behaviors were thus valued as positive and negative behaviors as negative by both participants in the interaction.

### Direct Effects of Interacting with a Depressed Partner

It was first examined what the direct effects of interacting with a depressed or non-depressed peer were on the nonverbal responses of adolescents. Means and standard deviations for nonverbal responses towards depressed and non-depressed adolescents are reported in Table 1. Interacting with a depressed peer predicted more negative facial expressions in the total sample (*B* = 0.57, β = 0.20 *p* < .001), as well as for both girls (*B* = 0.66, β = 0.23, *p* = .005) and boys (*B* = 0.46, β = 0.17, *p* = .013) separately. Additionally, interacting with a depressed peer also predicted more signs of discomfort for the total sample (*B* = 0.161, β = 0.12, *p* = .046) and for girls separately (*B* = 0.27, β = 0.19, *p* = .017).

Contrary to expectations, no direct effects of depression on observed personality traits were found (see Table 1), indicating that depressed and non-depressed adolescents did not differ in observed personality traits. Additionally, girls and boys did not differ in observed personality traits, contrary to what was expected. Girls and boys did show differences in nonverbal behaviors, as expected (see Table 1). Boys displayed significantly more negative nonverbal behaviors (negative facial expressions and disinterest) while girls displayed significantly more positive nonverbal behaviors (gazing while listening and speaking, smiling and backchanneling).

**Table 1:** Means and standard deviations (in parentheses) of adolescent’s nonverbal behaviors and observed personality expressed towards a (mildly) depressed versus nondepressed interaction partner

	Girls			Boys		
	Total	Nondepressed	Depressed	Total	Nondepressed	Depressed
*Nonverbal Behaviors*						
Neg. facial expressions	2.71 (1.39)^S^	2.41 (1.26)*	3.07 (1.55)*	3.43 (1.48)^S^	3.23 (1.37)*	3.69 (1.32)*
Disinterest	1.56 (0.61)^S^	1.36 (0.45)	1.42 (0.48)	1.86 (0.62)^S^	1.89 (0.57)	1.82 (0.69)
Discomfort	1.83 (0.63)	1.71 (0.67)*	1.98 (0.74)*	1.75 (0.56)	1.89 (0.58)	1.93 (0.63)
Gazing (listening)	54.61 (24.89)^S^	59.13 (24.81)	56.30 (25.32)	37.55 (26.26)^S^	38.23 (25.59)	34.82 (24.91)
Gazing (speaking)	33.14 (21.60)^S^	33.27 (21.58)	31.34 (22.04)	23.98 (22.55)^S^	29.57 (21.30)	26.38 (23.31)
Smiling	2.29 (1.19)^S^	2.34 (1.18)	2.23 (1.12)	1.91 (1.19)^S^	1.96 (1.29)	2.16 (1.50)
Back-channeling	4.98 (4.36)^S^	5.59 (6.02)	5.02 (2.97)	3.44 (3.80)^S^	3.67 (3.51)	3.20 (3.50)
*Observed personality of partner*						
Neuroticism	2.53 (0.90)	2.55 (0.85)	2.52 (0.95)	2.51 (0.99)	2.41 (0.91)	2.62 (1.07)
Agreeableness	3.07 (0.84)	3.07 (0.76)	3.07 (0.93)	3.10 (0.77)	3.21 (0.73)	3.00 (0.80)
Expressivity	2.63 (1.12)	2.57 (1.09)	2.68 (1.16)	2.72 (1.10)	2.54 (0.94)	2.90 (1.23)
Dominance	2.66 (0.98)	2.73 (0.92)	2.59 (1.04)	2.38 (0.92)	2.36 (0.87)	2.41 (0.97)

*Note* *= significant differences between nonverbal responses towards depressed or nondepressed partners (*p* < .05). ^S^ = significant differences in nonverbal responses between totals of girls and boys (*p* < .05)

### Mediation Analyses

The lack of direct effects between depression (0/1) and observed personality, indicated that there were no significant differences in any of the observed traits between depressed and nondepressed partners, contrary to expectations. Therefore, personality traits did not mediate the difference in peer responses towards depressed versus nondepressed partners.

### Moderation Analyses

In Table 2, the direct effect of interacting with a depressed partner (as compared to a non-depressed partner) on nonverbal peer responses, as well as the direct effect of the observed personality traits on nonverbal peer responses are reported; when the interaction term appeared significant, the direct and interaction effects from the moderation model are reported. As multi-level analyses were conducted per personality trait, the results are reported accordingly.

**Table 2:** Significant direct and interaction effects of observed personality traits and interacting with a depressed peer on nonverbal responses

	Total				Girls				Boys		
	Direct (dep.)	Direct (pers. trait)	Interaction		Direct (dep.)	Direct (pers. trait)	Interaction		Direct (dep.)	Direct (pers. trait)	Interaction
Neuroticism											
Neg. facial expressions	B = 0.50 β = 0.19	B = 0.30 β = 0.21			B = 0.67 β = 0.22	B = 0.41 β = 0.25			B = 0.41 β = 0.15		
Disinterest									B = 0.68 β = 0.54		B = -0.29 β = -0.69
Discomfort	B = -0.57 β = -0.43		B = 0.29 β = 0.64				B = 0.35 β = 0.71				
Gazing (speaking)						B = -5.30 β = -0.22					
Agreeableness											
Gazing (listening)									B = -56.66 β = -1.13	B = -12.12 β = -0.37	B = 16.92 β = 1.08
Gazing (speaking)									B = -48.67 β = -1.10	B = -10.20 β = -0.35	B = 14.46 β = 1.05
Expressivity											
Disinterest					B = 0.49 β = 0.53		B = -0.16 β = -0.54		B = -0.91 β = -0.73		B = 0.31 β = 0.82
Smiling		B = 0.18 β = 0.16									
Dominance											
Neg. facial expressions		B = -0.30 β = -0.20				B = -0.33 β = -0.23					
Gazing (speaking)						B = 4.55 β = 0.21					B = -8.33 β = -0.52

*Note*: Only significant effects are displayed in table (*p* < .05); In the case of non-significant interactions, direct effects are reported from the models specified without the interaction terms. In the case of significant interactions, direct effects are reported from the models specified with interaction terms added

#### Neuroticism

Neuroticism significantly predicted a greater amount of negative facial expressions in the total sample, and specifically for girls separately, indicating that the more neurotic an adolescent girl was observed to be, the more negative facial expressions they received from the adolescent peer they were interacting with. Only for girls, higher scores of observed neuroticism also predicted significantly less gazing while speaking from their peers.

Observed neuroticism moderated the relationship between interacting with a depressed partner and signs of discomfort, for the total sample, and particularly for girls. Adolescent peers, especially girls, displayed more signs of discomfort when interacting with a partner who was both depressed and observed to be neurotic, than a partner who was depressed but not observed to be neurotic.

For boys, no direct effects were found for observed neuroticism on nonverbal responses, contrary to the hypothesis. However, observed neuroticism did moderate the relationship between interacting with a depressed partner and signs of disinterest for boys. Adolescent boys displayed *more* signs of disinterest towards partners who were depressed but low in neuroticism, but when the depressed partners also were observed to be high in neuroticism this negative response was weakened.

#### Agreeableness

No direct or moderating effects were found for observed agreeableness in the total sample or for girls separately, contrary to the hypothesis that agreeableness would lead to more positive responses from girls. Only when specifying a moderation model that included both the direct and interaction effects, were several significant effects found for boys. For boys, when the interaction term was included, interacting with a depressed peer significantly negatively predicted gazing while listening, as well as gazing while speaking, indicating that boys gazed less at a depressed adolescent both while listening and speaking. Gazing while listening and gazing while speaking, were also significantly predicted by observed agreeableness for boys, in that the more agreeable a boy was observed to be, the less the peer gazed while listening and speaking. Additionally, agreeableness moderated the effect of interacting with a depressed boy on both gazing while listening and gazing while speaking. The negative response of less gazing towards agreeable boys was present when they were non-depressed, however when boys were observed to be agreeable *and* depressed, the effect of less gazing while listening and speaking was weakened.

#### Expressivity

For expressivity, it was found that a higher observed expressivity score led to more smiling from the peer in the total sample regardless of depression of the partner. When specifying the models separately for boys and girls, a difference was found when examining the moderation analyses for disinterest. When the interaction term of observed expressivity and interacting with a depressed peer was included, significant direct effects of interacting with a depressed peer were found. Girls displayed more signs of disinterest when interacting with a depressed peer and expressivity negatively moderated this relationship, indicating that the negative response towards a depressed girl was lessened when a girl was observed to have high expressivity, as expected. Boys however, displayed less signs of disinterest when interacting with a depressed versus nondepressed partner, although when the depressed partner was observed to be high in expressivity this difference was slightly weaker.

#### Dominance

Observed dominance was found to predict significantly fewer negative facial expressions for the total sample, and particularly for girls, indicating that the more dominant an adolescent girl was observed to be in interaction, the less negative facial expressions their interaction partner would display. For girls, observed dominance was also found to be a significant predictor of gazing while speaking, in that the more dominant a girl was observed to be, the more her interaction partner would gaze at her while speaking. For boys, observed dominance had no significant direct effects on the nonverbal responses of their peer partner, however, observed dominance did moderate the relationship between interacting with a depressed adolescent and gazing while speaking. Observed dominance was responded to with more gazing while speaking when the boy was not depressed but responded to with less gazing while speaking when the boy was depressed.

## Discussion

The present study was the first to examine the role of *observed* personality traits in the relationship between being mildly depressed (versus nondepressed) and negative nonverbal responses from peers in adolescence. It was investigated whether certain observed traits mediated the more negative peer responses towards (mildly) depressed adolescents (mediation model), or whether these traits moderated such responses.

It was confirmed that girls in general show more positive non-verbal behaviors and boys display more negative non-verbal behaviors than girls. Findings further confirm that indeed more negative social behaviors are shown when interacting with a (mildly) depressed adolescent than a non-depressed adolescent, in that more negative facial expressions and more signs of discomfort were displayed, the latter particularly by girls. This is in line with previous research that mild depression in adolescence is accompanied by more negative responses by peers [8, 9] and provides further support that subclinical depressive symptoms in a population sample may already lead to negative social consequences and erode social support [7].

For the main relationships between personality traits and nonverbal peer responses, as expected, higher neuroticism was found to predict more negative social responses for all adolescents, though especially for girls. Additionally, both expressivity and dominance, indicators of extraversion, were found to predict less negative social responses for girls. This is in line with literature demonstrating that extraversion and low neuroticism were associated with more likeability and popularity [26]. The present study did not show that agentic traits, especially dominance, are specifically more positively regarded for boys [34]. When considering the interaction effect, dominance did reveal to elicit more positive responses but then only for non-depressed boys. Contrary to our expectations, agreeableness did not predict less negative or more positive responses. Descriptive analyses demonstrated that little variance was found for agreeableness, in that adolescents rarely demonstrated very disagreeable behavior during the observations. A possible explanation is that the setting, a videotaped interaction, could very well have inhibited adolescents from demonstrating overt argumentativeness, as they were aware they were being observed. For future research using a similar design, a more specified scale could be used that is able to distinguish to a greater degree subtle differences in agreeableness even in short observed interactions.

When examining the relationships between being depressed or non-depressed and observed personality, no significant direct relationships were found in the mediation model, contrary to our expectations and a large body of literature indicating strong relationships between depression and extraversion and neuroticism [18, 47, 48]. It may be that associations between depression and personality are more distinct when examining clinical depression and are not yet as apparent when examining depressive symptoms in a population sample. Moreover, the present study examined personality through observations, as opposed to the more common self-reports. Perhaps the link between personality and depression is in the strong cognitive aspects of personality, such as cognitive strategies or interpretation of loci of control, which are more readily measured through self-reports and are less observable in interaction. The expression of personality traits through behaviors may be less stable during adolescence, and it is important to investigate whether observed personality and self-reports are indeed highly correlated or even associated during this period. Considering that no group differences were found between being depressed or non-depressed in personality traits, we can conclude that observed personality traits did not mediate the more negative responses towards (mildly) depressed adolescents.

Several striking results were found when examining if personality traits strengthen or weaken certain social responses to depressed adolescents. In line with the expectations, it was found that for girls, as well as in the total sample, observed neuroticism did strengthen the negative social responses to depressed adolescents. Confirming that neuroticism is contrary to social expectations in interaction, thereby making their interaction partner feel greater discomfort [26]. When girls were observed to have higher neuroticism as well as depression, the responses from their peers were even more negative. For boys, however, only when taking neuroticism into account, did interacting with a depressed adolescent predict signs of disinterest, but observed neuroticism *weakened* this response This is a rather counterintuitive result, considering both neuroticism and depression are generally responded to negatively. It may be that the behaviors that depressed boys display in interaction might be interpreted differently when they are also observed to be more neurotic, e.g., as being insecure or nervous as opposed to being disinterested or uninvolved. Alternatively, it may be that depressed boys display fewer behaviors in interaction, thereby being less interesting, but that neuroticism compensates for this, through nervous or tensed behaviors, increasing the interest of their interaction partner. More clarity is needed regarding the relationship between observed personality traits and associated behaviors in boys and girls, as well as how male and female peers value and respond to these (combination of) behaviors.

Agreeableness did not display any direct effects on interacting with a depressed adolescent. That agreeableness did not seem to play a moderating role in general, nor for girls may be explained by the lack of variance (no low scores) in agreeableness due to applying to social expectations in this particular videotaped situation (see Table 1). Agreeableness had a high mean indicating that they may have all acted relatively agreeable, as expected from them in social situations. Only when examining a model with interaction terms and for boys alone, did agreeableness reveal to play a role; high agreeableness in boys evoked more negative responses from the peer, though these responses were weakened if the boy was (mildly) depressed. This seems a rather counterintuitive result and merits further behavioral research to examine whether this finding is confirmed when there is more variance in agreeableness and to clarify which characteristics are displayed during interaction that are indicative for depression and/or agreeableness.

Expressivity played a protective role for girls by reducing the negative response, however for boys expressivity led to a greater negative response. Dominance similarly strengthened the negative response towards depressed boys. It should be investigated if the nature of the expressivity and dominance was more negative in depressed boys, for instance through provocative and irritating dominant behaviors or more negative emotions, as opposed to more supportive or constructive dominant behaviors. The findings concerning expressivity and dominance together seem to indicate that extraversion will protect depressed girls from the negative social responses, but will increase the negative social responses towards depressed boys.

Though this study is unique in examining the interaction between personality, as observed in behavior, depression and peer responses in terms of specific nonverbal behaviors, some caution is warranted. As with many studies using observation data, power was limited by a relatively small sample size. Some effects may be situation-specific, while other (smaller) effects that may actually occur in reality were not found. Analyses were conducted for boys and girls separately, the moderation results that were found within those models but not within the general sample may be less reliable and definitely warrant further research. Future studies would do well to examine similar relationships in larger samples and more natural settings, to confirm the results. Another limitation of the present study is that only a limited number of nonverbal responses were taken into account. Though the present study strove to include most nonverbal behaviors used in earlier literature [9, 42], there are still behaviors or other characteristics, such as intonation, physical appearance or the verbal content being voiced to depressed adolescents that may strengthen the results found. A strength is that the observed nonverbal behaviors used in the present study did correlate positively to the evaluation of the quality of the interaction as the preliminary analyses demonstrate.

It was beyond the scope of the present study to examine what combinations of behaviors are characteristic of depressed boys and girls with certain (combinations of) personality traits, and how these differ from non-depressed adolescents. Building on this, sequential analyses could be conducted to examine how differing combinations of personality traits and associated nonverbal behaviors elicit different responses from peers. Another limitation is that effects of personality traits were analyzed separately, as testing them together was not possible with this sample size. Stronger or differing relationships may be found if personality could be examined using, for instance, latent classes. A final limitation is that personality traits were observed and coded by graduate students. This was to ensure that the same construct was examined and reliably coded. However, it remains unknown whether adolescents observe personality traits among peers in a similar manner and if there may be other characteristics, in addition to the personality traits observed in this study, that adolescents might find important and respond to during interaction. It would also be beneficial to examine these relationships in real-life interactions or in comparison with personality questionnaire data, as well as to examine how stable observed personality traits remain over the years.

### Summary

The present study found that the behavioral characteristics adolescents show when they are (mildly) depressed do indeed lead to more negative peer responses, as found in previous research [8, 9], but that this association can be strengthened or weakened by observed personality traits dependent on gender. Examining the nonverbal responses towards personality traits as observed in behavior, adds to and supports the literature that almost exclusively focuses on perceptions of popularity, likeability, or satisfaction of interactions. Even without clear differences between (mildly) depressed and nondepressed adolescents in observed personality traits, these traits still strengthened, weakened, or reversed peer responses towards depression dependent of gender. Clinicians need to take personality, and how it is expressed or seen in social behavior, into account in both intervention and prevention. Adolescents can be made aware of the impression others are observing of them through their behaviors and be shown how this might result in certain responses. Preventive clinical efforts may also focus on adolescents, especially girls who display high neuroticism, and train them in social skills or cognitive restructuring to avoid the exacerbating effects of neuroticism on responses towards depressed adolescents [49]. Both clinical practice and research should keep into account that though observed personality does influence the relationships, it is *not* the underlying characteristic that explains the negative responses towards depressed adolescents. This begs the question: what other characteristics do (mildly) depressed adolescents display in interaction that may explain the negative peer responses that already seem to exist prior to the development of depressive symptoms and also remain after a depressive episode, increasing the risk for recurrence in adulthood [50–53]? Research should further examine possible behaviors or characteristics of depressed adolescents that may play a role, and which of these behaviors or characteristics specifically trigger the negative responses, for instance through sequential analysis of the behaviors displayed by both adolescents in interaction and by investigating how these behaviors may relate to social or generalized anxiety for instance, which have been shown to predict depression [54]. Additionally, it is important to examine which behaviors cause certain personality traits to be observed during interaction. If these predictors in interaction can be identified, clinicians can help adolescents to adapt those behaviors that may trigger a certain perception and negative social response in peers.

## Electronic supplementary material

Below is the link to the electronic supplementary material.

Supplementary Material 1

## Data Availability

Access to datasets and materials may be requested from authors Marry Schreur and Yolanda van Beek.
