# Supplementary material for: How Observed Personality Traits in (Mildly) Depressed Adolescents Relate to Nonverbal Responses of Peers
Source: Child Psychiatry Hum Dev. 2024 Feb 12;56(6):1670–81. doi: 10.1007/s10578-024-01669-3 (PMC12628427; doi:10.1007/s10578-024-01669-3)
Supplement: Supplementary file 1 — Supplementary Material 1 [file 10578_2024_1669_MOESM1_ESM.docx]

**Appendices (submitted as related information)**

**Title**: How Observed Personality Traits in (Mildly) Depressed Adolescents Relate to Nonverbal Responses of Peers.

**Journal**: Child Psychiatry & Human Development

**Author information**

Marry Schreur, MSc., Department of Developmental Psychology, Faculty of Social and Behavioral Sciences, Utrecht University, Utrecht the Netherlands. ORCID: 0000-0003-0408-2499

Dr. Yolanda van Beek, Department of Developmental Psychology, Faculty of Social and Behavioral Sciences, Utrecht University, Utrecht the Netherlands. ORCID: 0000-0001-9986-9467

Dr. R. Hutteman, Department of Developmental Psychology, Faculty of Social and Behavioral Sciences, Utrecht University, Utrecht the Netherlands.

**Appendix A.** Description of Nonverbal behaviors as coded in observation

*Note to editor/reviewer*: Appendix is added as additional information for reviewers, not essential for publication. Information is also available upon request from authors.

**Other-oriented behaviors**

These behaviors were coded using the Observer XT program (Noldus Information Technology, 2015). Behaviors are scored as they occur, and are recorded on the millisecond. Coders indicate as soon as a specific behavior starts or stops.

*Gazing:* when participant is looking directly at the face of their interaction partner

- Interruptions in gazing of 1 second or less do not need to be coded.
- Gazing while listening = percentage of gazing while interaction partner is talking
- Gazing while speaking = percentage of gazing while participation is talking

*Smiling:* when the participant smiles or laughs

- Clear happy facial expression, not scored when participant is talking themselves, unless there is laughter between the talking.

*Back-channeling:* only when the participant responds affirmatively to the talking of the interaction partner, without interrupting them, by nodding or making an affirmative noise

- It is only scored as a second backchannel if the nodding is interrupted (min. 1 sec). As long as the nodding continues, it is scored as the same backchannel.

**Negative behaviors**

Table 3

*Behaviors and rating scales of clusters of negative behaviors*

| *Negative facial expressions* | Frowning  Skin folds  Pressed lips  Puffing the cheeks  Pursed mouth  Yawning  Raising eyebrows  Closing eyes  Staring into the distance  Rubbing eyes  Expressionless face | 1. 0-4 times / less than 20%  2. 5-9 times / 20-30%  3. 10-12times / 30-50%  4. 13-15 times / 50-70%  5. 16 times or more / 70% or more |
| --- | --- | --- |
| *Signs of Disinterest* |  |  |
| Disinterested/bored  behaviors | Checking watch  Head on elbows  Shrugging shoulders  Sighing  Chewing gum | 1. 1-2 times  2. 3-4 times  3. 5-7 times  4. 8-10 times  5. 11 times or more |
| Disinterested/bored posture | Slouching  Hands in pockets | 1. 0-20% of the time  2. 20-40% of the time  3. 40-60% of the time  4. 60-80% of the time  5. 80-100% of the time |
| Intonation | Monotonous speech | 1. 0-30% of the time  3. 40-60% of the time  5. 70-100% of the time |
| *Signs of Discomfort* |  |  |
| Nervous body touching | Touching hair  Hands-to-face  Hands-to-mouth  Scratching  Pulling on clothes  Rubbing eyes | 1. 0-4 times / less than 20%  2. 5-9 times / 20-30%  3. 10-12times / 30-50%  4. 13-15 times / 50-70%  5. 16 times or more / 70% or more |
| Tensed body position | Chin towards chest  Bending over  Stiff/tense posture | 1. 0-2 times / less than 10%  2. 3-4 times / 10-20%  3. 5-7 times / 20-40%  4. 8-10 times / 40-60%  5. 11 times or more / 60% or more |
| Facial restlessness | Rubbing lips  Biting on lips  Biting on cheeks  Pulling mouth back | 1. 0-2 times / less than 10%  2. 3-4 times / 10-20%  3. 5-7 times / 20-40%  4. 8-10 times / 40-60%  5. 11 times or more / 60% or more |

**Appendix B.** Description of Personality traits as rated in observed conversations.

*Note to editor/reviewer*: Appendix is added as additional information for reviewers, not essential for publication. Information is also available upon request from authors.

**Neuroticism:** is characterized by proneness to anxiety, emotional instability, self-consciousness, and over-sensitivity. They are fearful, nervous, and unconfident. People with a low score in neuroticism are emotionally stable, peaceful, unflinching (McCrae & Costa, 1987; Goldberg, 1990).

**How neurotic** **do you perceive person A/B to be in this conversation?**

1. Not neurotic
2. A little neurotic
3. Relatively neurotic
4. Quite neurotic
5. Very neurotic

**Agreeableness:** the measure of good-naturedness, kindness, and warmth a person displays. It is characterized by cooperativeness, friendliness, patience, trustworthiness and politeness.

A person with a low score of agreeableness is argumentative, doesn’t display warmth or kindness (McCrae & Costa, 1987; Goldberg, 1990).

**How agreeable do you perceive person A/B to be in this conversation?**

1. Disagreeable
2. Not disagreeable/not agreeable
3. A little agreeable
4. Relative agreeable
5. Very agreeable

**Expressivity**: being able to express, not only with words, what one means or how one is feeling. They are active, demonstrative, communicative and energetic in their mannerisms. A person with low expressivity is passive and displays little action or emotion (McCrae & Costa, 1987; Goldberg, 1990).

**How expressive do you perceive person A/B to be in this conversation?**

1. Unexpressive
2. Neutral
3. A little expressive
4. Relatively expressive
5. Very expressive

**Dominance**: being dominantly present during conversation. It is characterized by being talkative and verbal, assertive, and confident. They can be loud and forceful. A person with low dominance is characterized by being not dominant, reserved, and unassuming (McCrae & Costa, 1987; Goldberg, 1990).

**How dominant do you perceive person A/B to be in this conversation?**

1. Not dominant
2. Neutral
3. A little dominant
4. Relatively dominant
5. Very dominant
